# Structural Features and Transcriptional Activity of Chicken PPARs (**α**, **β**, and **γ**)

**DOI:** 10.1155/2013/186312

**Published:** 2013-01-03

**Authors:** Ichiro Takada, Mime Kobayashi

**Affiliations:** ^1^Department of Cell and Tissue Biology, School of Medicine, Keio University, Tokyo 160-8582, Japan; ^2^Department of Microbiology and Immunology, School of Medicine, Keio University, Tokyo 160-8582, Japan; ^3^Graduate School of Biological Sciences, Nara Institute of Science and Technology, Nara 630-0192, Japan

## Abstract

While an understanding of lipid metabolism in chickens is critical for a further improvement of food production, there are few studies concerning differences in lipid metabolism mechanisms between chickens and other species at a molecular level. Chickens have three PPAR gene subtypes (**α**, **β**, and **γ**) that function differently from those present in humans and mice. The chicken PPAR-gamma (cPPAR**γ**) gene is shorter than that in humans and lacks a **γ**2 isoform. Moreover, in serum-free media, cPPAR**γ** shows high transcriptional activity without exogenous ligands. Luciferase reporter assays were used to examine the effect of sera on cPPAR transcriptional activities and showed that adult bovine serum and chicken serum highly activate cPPAR**α** and **β** functions. Moreover, we found that bezafibrate induces the transactivation function of cPPAR**β**, but not human PPAR**δ** (human PPAR**β** ortholog). This ligand selectivity relies on one amino acid residue (chicken: Val419, human: Met444). These results show the possibilities for unique functions of cPPARs on chicken-specific lipid glucose metabolism. As such, a better understanding of the molecular mechanisms of lipid metabolism in chickens could result in higher productivity for the poultry industry.

## 1. Genomic Organization of Chicken PPARS (cPPARs)

The mechanisms of lipid and glucose metabolism for energy storage and homeostasis differ between species in ways that are not fully understood. To improve food production, comprehending the molecular basis for lipid and glucose metabolism in domestic animals is a significant issue. In avian species, lipids, especially triglycerides, are thought to be stored in adipocytes, hepatocytes, and growing oocytes. Excessive accumulation of lipids in the adipose tissue of broilers is a major concern for producers, because excessive fat deposits result in lower meat yields.

**Figure 1:**
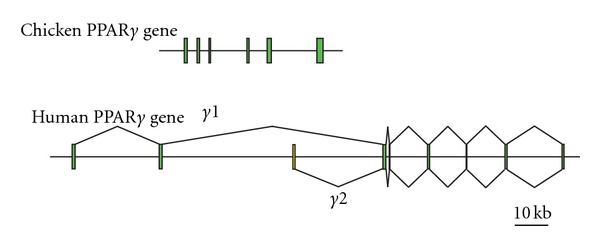
Structural organization of chicken (upper panel) and human (lower panel) PPAR*γ* genes. Exons are shown as green boxes with the *γ*2-specific exon colored in yellow.

In 2000, we reported the cloning of three chicken PPAR subtype genes (*α*, *β*, and *γ*) from cDNA libraries prepared from 2.5-day-old chicken embryos and adult adipose tissue [1]. For cPPAR*γ*, amino acid residues predicted for phosphorylation by MAP kinase [2] (Ser82 in cPPAR*γ*) and Cdk5 [3] (Ser243 in cPPAR*γ*) were conserved between mammals and chicken PPAR*γ*. Moreover, predicted SUMOylation target residues (Lys77 and Lys365 in cPPAR*γ*) were also conserved in cPPAR*γ* [4]. This shows the possibility that the transcriptional activity of cPPAR*γ* regulates growth factors and that cPPAR*γ* activation has an anti-inflammatory effect. However, we were unable to isolate the PPAR*γ*2 splicing variant identified in mammals [5, 6]. This variant is likely absent in chickens because Sato et al. also could not isolate chicken PPAR*γ*2 by 5′-RACE [7] and because no sequence similar to a PPAR*γ*2 isoform has been found in the chicken genome database (Figure 1). The PPAR*γ*2 isoform has not been reported in *Xenopus* and zebrafish either. Furthermore, while the structural organization of PPAR*α* and *β*/*δ* genes is largely conserved from humans to chickens, the PPAR*γ* isoform is not. The cPPAR*γ* gene (Ensembl ID: ENSGALG00000004974) has 6 exons and extends across ~50 kilobases (kb) of genomic DNA (Figure 1, upper panel). In contrast, the human PPAR*γ* gene (Ensembl ID: ENSG00000132170) consists of 8 and 7 exons for the *γ*1 and *γ*2 subtypes, respectively, and extends across more than 160 kb of genomic DNA (Figure 1, lower panel). Other species such as squirrel (Ensembl ID: ENSSTOG00000012778), hedgehog (ENSEEUG0000006334), Chinese softshell turtle (ENSPSIG00000011100), and Anole lizard (ENSACAG00000013360) have the chicken-type organization of PPAR*γ* genes. 

 In mammals, several polymorphisms of PPAR genes have been associated with metabolic disorders. In chicken, single nucleotide polymorphism (SNP) analysis has been conducted to find the association with lean and fat broilers [8]. In this study, PPAR*γ* c-75G>A is reported to be associated with abdominal fat weight and also correlates with SNPs of other lipid metabolism-related genes (Fsbp4, C/ebp*α*, etc.).

Promoter analysis of the cPPAR*γ* gene revealed that transcription factor binding sites (such as C/EBP*α*, Sp1 and AP1) exist as is the case with mammalian PPAR*γ*1 and PPAR*γ*2 promoters. Interestingly, structure of cPPAR*γ* promoter is more similar to the mammalian PPAR*γ*2 promoters compared to PPAR*γ*1 promoters [9]. These results suggest that C/EBP*α*-PPAR*γ* pathway is conserved across the species and that PPAR*γ*2 gene first appeared in the course of evolution when additional PPAR*γ* isoforms have been acquired.

## 2. Expression Patterns of cPPAR mRNAs

 The mRNA expression patterns of cPPARs are similar to rodents and primates. In adult chicken, cPPAR*α* is highly expressed in the heart, liver, and kidney [10]. As is the case for PPAR*β* or *δ* in other species, cPPAR*β* is expressed ubiquitously. Meanwhile, high expression of cPPAR*γ* is observed in fat tissue as well as the kidney, heart, brain, liver, and intestine [7, 11]. Recent studies using renal-specific PPAR*γ* knockout mice have shown that the gene regulates body weight by controlling water channel activities [12]. Thus, cPPAR*γ* may also regulate body weight in chickens.

 Hojo et al. examined cPPAR mRNA expression patterns in digestive organs during embryonic development in chickens [13]. In embryonic day 6 (E6), E9, and E12 embryos, PPAR*α* is expressed in the epithelium, smooth muscle, and small intestine. PPAR*β* is expressed in the epithelium and moderately in mesenchymal tissue on E6, E9, and E12. PPAR*γ* is weakly expressed in the epithelium on E9 and E12 and expressed in the medial and basal parts of the small intestine villi, the villi of PH3 and 14, and the crypt epithelium of the large intestine. 

## 3. Regulators of cPPAR mRNAs 

 PPAR mRNA levels are known to be regulated in aging, metabolic disorder, and cancer [14]. In general, mRNA levels of PPARs or their target genes are upregulated in obesity because of increased fatty acids. Several regulators of PPAR mRNAs have recently been identified in chicken.

 In adipocyte, accumulation of nonesterified fatty acids (NEFA) induces cPPAR*γ* mRNA, and a cPPAR*γ* downstream gene (adipocyte fatty acid binding protein (A-FABP)) controls the concentration of lipid and NEFA [15]. In fatty broiler chickens, cPPAR*γ* mRNA expression is induced in liver but not in adipose [16]. Because PPAR*γ* mRNA is induced in adipose tissue of obese mammals [17, 18], the result indicates a divergence of PPAR signal transduction mechanisms between avians and mammals.

 Regulation of cPPAR*α* mRNA is also reported. Comparison of gene expression profiles in the intramuscular fat of two broiler lines showed that PPAR*α* and MAPK pathway are enhanced in both Beijing-you (BJY), which is a slow-growing Chinese breed possessing high meat quality, and Arbor Acres (AA), a commercial fast-growing broiler line [19]. Upon feed restriction, mRNA expression levels of cPPAR*α* and one of its target gene, peptide transporter 1 (PepT1), are induced in the jejunum [20]. 

 In myoblasts, isoproterenol (a *β*-adrenergic receptor agonist) and fatty acid enhance the phosphorylation of cPPAR*α*. The mRNA of UCP3 is induced through cPPAR*α* and AMPK-PKA pathway [21]. In spleen, cPPAR*α* and *γ* mRNAs are induced by lutein, a plant carotenoid, and reduced by lipopolysaccharide (LPS) [22]. These results indicate that some regulators of cPPARs mRNA expression are conserved between chicken and mammals.

## 4. Comparison of the Amino Acid Sequences between Chicken and Mammalian PPARs

 The amino acid sequence in the DNA binding domain (DBD) of cPPARs shows high similarity with mammals (cPPAR*α* versus hPPAR*α*: 95%; cPPAR*β* versus hPPAR*δ*: 98%; cPPAR*γ* versus hPPAR*γ*: 100%). The similarity of the ligand binding domain (LBD) is also high for PPAR*α* and PPAR*γ* (cPPAR*α* versus hPPAR*α*: 96%; cPPAR*γ* versus hPPAR*γ*: 96%). However, the LBD of cPPAR*β* shows lower similarity between chicken and humans (90%), mice (86%), and *Xenopus* (78%). Because this diversity implies different ligand responses, we conducted luciferase reporter assays to examine the ligand-inducible transcriptional activity of cPPARs.

## 5. Transcriptional Activities of cPPARs

 We first generated cDNA expression vectors of cPPAR LBDs fused to GAL4 DBD. The ligand-inducible transcriptional activities of them were examined by luciferase reporter assays. We used serum-free conditions ((DMEM/ITB; DMEM with 5 *μ*g/mL insulin, 5 *μ*g/mL transferrin, and 0.01% fatty acid-free bovine serum albumin (BSA)) to reduce the effect of lipids contained in serum.

 Rhesus monkey kidney CV-1 cells cultured in 24-well cluster tissue culture plates were transfected with expression vectors carrying GAL4-cPPAR LBDs (25 ng/well), pCMX-*β*GAL (100 ng/well, as a control for transfection efficiency), and the MH100X4-tk-luc reporter plasmid (150 ng/well) using calcium phosphate precipitation method [23].

**Figure 2:**
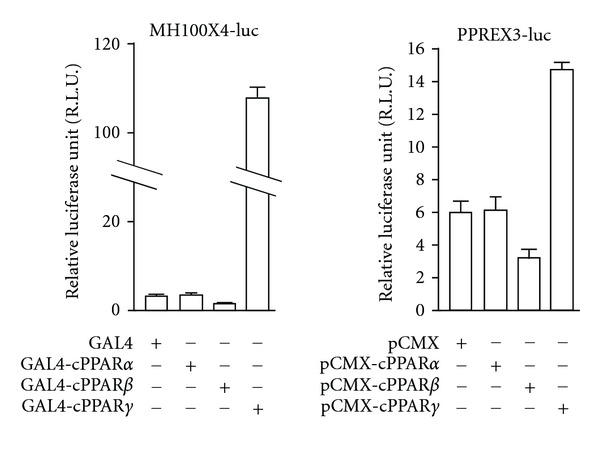
Transactivation function of cPPARs in serum-free media. After transfection with each plasmid mixture, cells were cultured for 36 hrs, lysed, and luciferase reporter assays were performed. MH100X4-luc is a GAL4 responsive luciferase reporter, and PPREX3-luc is a PPAR responsive luciferase reporter. Data are derived from triplicate points from two independent experiments and represented as the mean ± SE; *n* = 6. The results presented in this figure have been published [1].

 Interestingly, GAL4-cPPAR*γ* LBD showed high transcriptional activity without exogenous ligands (Figure 2, left panel). This activation was also observed for full-length cPPAR*γ* (Figure 2, right panel), indicating that this activation is cPPAR*γ* dependent. Moreover, cPPAR*β* suppressed basal transcriptional activity without ligands in DMEM/ITB (Figure 2, both panels). These findings show the possibility of a distinct ligand-independent transactivation function of cPPARs.

**Figure 3:**
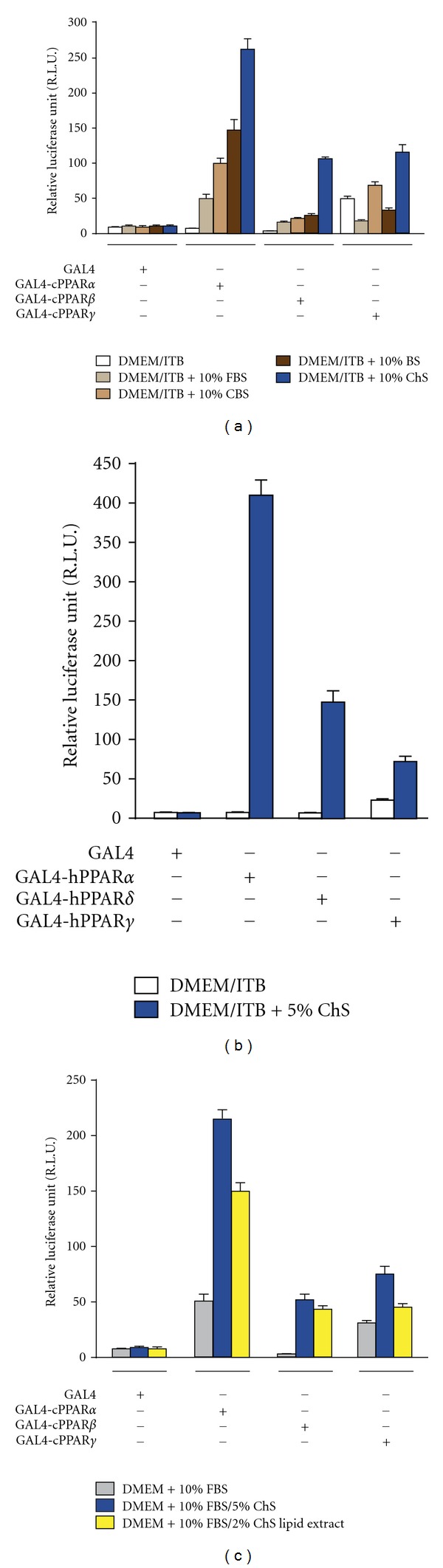
Effect of serum on the transactivation function of cPPARs. (a), (b). Effects of serum on the transactivation function of PPARs. Serum-induced transactivation function of cPPARs (a) or hPPARs. (b) After transfection with each plasmid mixture, cells were cultured with indicated serum conditions for 36 hrs, then lysed, and luciferase reporter assays were performed as described previously [1]. FBS: fetal bovine serum, CBS: calf bovine serum, BS: adult bovine serum, and ChS: chicken serum. (c) The effect of lipid extract from chicken serum (ChS) on the transactivation function of cPPARs. After being transfected with each plasmid mixture, cells were cultured for 36 hrs in DMEM/10%FBS with or without 5% ChS or ChS lipid extract equivalent of 2% ChS. Then cells were lysed and luciferase assays were performed. Data are derived from triplicate points from two independent experiments and represented as the mean ± SE; *n* = 6.

 We next investigated the effect of serum on the transcriptional activities of cPPARs. It was found that adult bovine serum induces cPPAR*α* transcriptional activities to a greater extent than fetal bovine serum and calf serum (Figure 3(a)). Interestingly, chicken serum (ChS) highly potentiated the transcriptional activities of cPPAR*α* and *β* as compared to bovine serum. ChS also activated the transactivation function of hPPARs (Figure 3(b)). Furthermore, using lipid extracts from chicken serum by a previously established method [24], we showed that the lipid extracts retain the enhancement effects of chicken serum for the transcriptional activities of cPPAR*α* and *β* (Figure 3(c)).

 These results show that serum from adult animals (chicken and bovine) contains more endogenous PPAR*β* ligands than that from fetal or young animals (bovine). Although the identity of PPAR*β* ligand(s) in adult serum has not yet been revealed, fatty acids and their derivatives are strong candidates.

## 6. Bezafibrate Is a Ligand for Avian and *Xenopus *PPAR***β***

 We next examined the effect of known PPAR activators (including fibrates and thiazolidinediones) on the transactivation function of cPPARs. Bezafibrate, a known ligand for *Xenopus* PPAR*β* (xPPAR*β*) [25], enhanced the ligand-inducible transactivation function of xPPAR*β* in our hands (data not shown) and cPPAR*β*, but not hPPAR*δ* [1].

**Figure 4:**
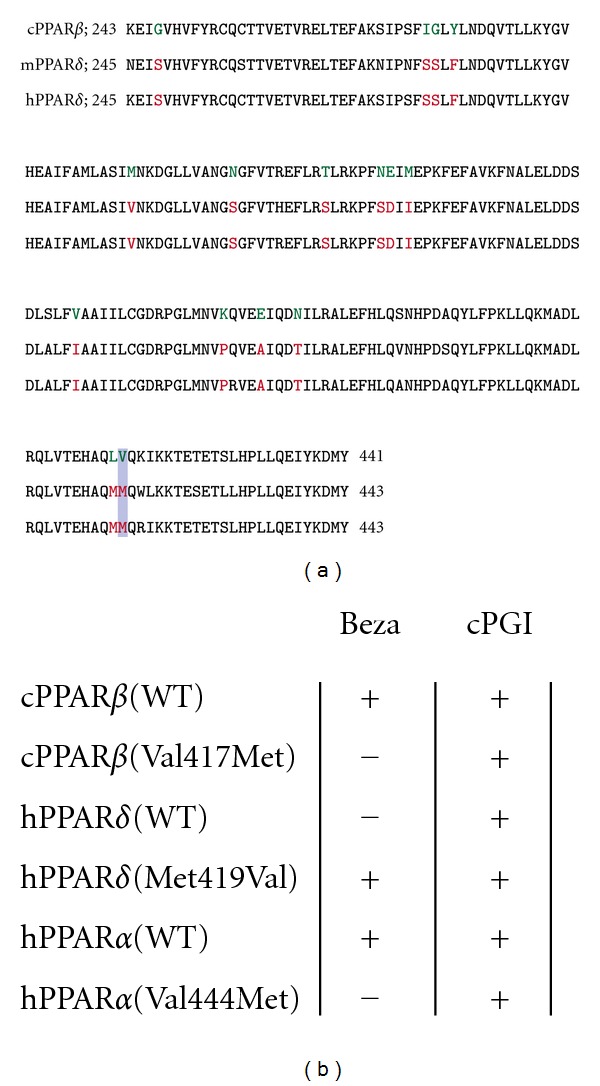
(a) Alignment of amino acid residues of chicken, mouse, and human PPAR*β*/*δ* LBDs. LBD is located at the C-terminus of chicken (amino acid residues 243 to 441) and mammalian (245 to 443) PPAR*β*/*δ* proteins. Amino acid residues conserved between mouse and human but not in chicken are highlighted in orange (mammals) and green (chicken). The amino acid residues critical for bezafibrate-dependent response of PPAR*β*/*δ* are indicated by a purple box. Accession numbers are as follows: cPPAR*β*, AAF80480; mPPAR*δ*, NP_035275; hPPAR*δ*, NP_001165289. (b) Bezafibrate (Beza) and carbaprostacyclin (cPGI) responsiveness of point mutated chicken PPAR*β*(Val419Met), human PPAR*α*(Val444Met), and PPAR*δ*(Met417Val) as previously reported [1].

 This result shows that bezafibrate selectively activates cPPAR*β* despite the relatively high structural similarity between cPPAR*β* and hPPAR*δ* LBDs. We looked for the amino acids that are conserved between human and mouse PPAR*δ*, but not chicken PPAR*β* and found that 15 amino acid residues were altered between chicken and mammals (Figure 4(a)). To identify the amino acid residue(s) critical for bezafibrate-dependent activation, we generated cDNA expression vectors carrying point mutations of relevant hPPAR*δ* and cPPAR*β* residues. Luciferase reporter assays in CV-1 cells were performed, and it was found out that a single amino acid alteration in cPPAR*β*(Val419Met0029 abrogates bezafibrate-dependent activation. When the corresponding Met residue in hPPAR*δ* was changed to Val, the mutant, hPPAR*δ*(Met417Val) enhanced bezafibrate-dependent activation. Human PPAR*α* has a Val residue at this position and when a point mutation was introduced, hPPAR*α*(Val444Met) abrogated bezafibrate responsiveness (Figure 4(b)). However, another PPAR*β*/*δ* activator, carbaprostacyclin, activates both hPPAR*δ*(Met417Val) and cPPAR*β*(Val419Met). These results suggest that the cPPAR*β* gene evolutionarily changed into the PPAR*δ* type, which is restricted to fibrate responsiveness. Although distinct endogenous ligands for chicken PPAR*β* and human/mice PPAR*δ* have not been identified, this amino acid change may reflect the difference between chicken and mammals in the regulation of energy storage [26].

 Moreover, identification of amino acid residues involved in ligand interactions will help to generate PPAR*δ*-specific ligands and facilitate the development of drugs to treat metabolic syndromes. Thus, investigation of species-selective PPAR ligand activity is significant for understanding species-specific lipid metabolism and development of new drugs.

## 7. Effects of Fibrate on Hens

 There are few *in vivo* analyses of chickens fed with PPAR ligands. A previous report showed that hens treated with clofibrate (cPPAR*α* ligand) had lower food intake and lost body weight during the 5-week feeding period, which in turn reduced egg production due to the absence of large follicle(s) and the presence of few small yellow and white follicles in the ovary [27]. Moreover, triglyceride levels in the livers of clofibrate-treated hens were reduced and mRNA levels of known PPAR*α* target genes such as Acyl-CoA oxidase (ACO), hepatic lipase, and lipoprotein lipase (LPL) were induced. In contrast, mRNAs of insulin-induced genes (Insig-1, SREBP-2, FAS, HMG-CoA reductase, and LDL receptor) were downregulated by clofibrate treatment. These results show that, as with mice and humans, cPPAR*α* regulates lipid metabolism in the liver and controls appetite [28].

## 8. Conclusions

 In this paper we discussed the properties of chicken PPARs based on their genomic structural organization and ligand-inducible and -independent transactivation functions. Evolutionary changes in the PPAR*γ* genomic structure may be one critical factor for species-dependent lipid homeostasis. Because there are few reports concerning the promoter activity of PPAR*γ* gene in chickens and other nonmammals, more detailed analyses will be required. Interestingly, the ligand-inducible transcriptional activities of cPPARs do differ from mammalian PPARs. Because a single amino acid change leads to a selective ligand response, the spectrum of endogenous ligands of PPARs in avian species may be different from that of mammals. As such, further analysis will be required to understand the comparative endocrinology and this information will be useful for industrial applications in the poultry and other food industries.
